# Attentional Biases and Their Association with Substance-Use-Related Problems and Addictive Behaviors: The Utility of a Gamified Value-Modulated Attentional Capture Task

**DOI:** 10.1016/j.abrep.2024.100534

**Published:** 2024-02-13

**Authors:** René Freichel, Erynn Christensen, Lana Mrkonja, Peter J. de Jong, Janna Cousijn, Ingmar Franken, Murat Yücel, Rico Lee, Ilya M. Veer, Lucy Albertella, Reinout W. Wiers

**Affiliations:** a Addiction Development and Psychopathology (ADAPT)-lab, Department of Psychology, University of Amsterdam, Amsterdam, the Netherlands; b BrainPark, Turner Institute for Brain and Mental Health and School of Psychological Sciences, Monash University, Clayton, Australia BrainPark, Clayton, Australia; c Department of Clinical Psychology and Experimental Psychopathology, University of Groningen, Groningen, the Netherlands; d Center for Substance Use and Addiction Research (CESAR), Department of Psychology, Education & Child Studies, Erasmus University Rotterdam, Rotterdam, the Netherlands; e QIMR Berghofer Medical Research Institute, Brisbane, QLD, Australia; f Melbourne School of Psychological Sciences, University of Melbourne, Parkville, VIC, Australia; g Center for Urban Mental Health, University of Amsterdam, Amsterdam, the Netherlands

**Keywords:** Addictive behaviors, Attentional bias, Cognitive control, Substance use

## Abstract

**Background:**

Attentional biases towards reward stimuli have been implicated in substance use-related problems. The value-modulated attentional capture (VMAC) task assesses such reward-related biases. The VMAC task widely used in lab studies tends to be monotonous and susceptible to low effort. We therefore tested a gamified online version of the VMAC that aimed to increase participant engagement. Our goal was to examine how VMAC is associated with substance use-related problems and addictive behaviors, and whether this association is moderated by cognitive control.

**Methods:**

We recruited 285 participants from an online community, including heavy alcohol users. All participants completed a novel gamified version of the VMAC task, measures of substance use and addictive behaviors (addictive-like eating behavior, problematic smartphone use), the WebExec measure of problems with executive functions, and the Stroop Adaptive Deadline Task (SDL) as a measure of cognitive control.

**Results:**

The gamified VMAC task successfully identified value-modulated attentional capture effects towards high-reward stimuli. We found no significant associations between VMAC scores, problematic alcohol or cannabis use, addictive behaviors, or any moderation by a behavioral measure of cognitive control. Exploratory analyses revealed that self-reported cognitive problems were associated with more alcohol-, and cannabis-related problems, and addictive behaviors. Greater attentional capture (VMAC) was associated with more cannabis use-related problems among individuals with higher levels of self-reported cognitive problems.

**Conclusions:**

Our study is one of the first to demonstrate the utility of the gamified version of the VMAC task in capturing attentional reward biases. Self-reported problems with cognitive functions represent a key dimension associated with substance use-related problems and addictive behaviors.

## Highlights

• We introduced a gamified value-modulated attentional capture (VMAC) task.
• This gamified task successfully captured VMAC effects toward high-reward stimuli.
• No associations between VMAC and problematic substance use or addictive behaviors.
• Self-reported cognitive problems were associated with all addictive behaviors.

## 1 Introduction

The past decades of research in cognitive science have established that attentional selection can be automatically influenced by both the physical salience as well as the reward value of stimuli (Anderson, 2021, Anderson et al., 2011, Le Pelley et al., 2015). The phenomenon of ‘sign tracking’ has been extensively observed in animals and it speaks to the ability of reward-predictive cues to capture attention (Colaizzi et al., 2020). Attention is more likely to be directed towards stimuli signaling high rewards, even in situations during which the reward-related stimulus is task-irrelevant or counterproductive (Pearson et al., 2022). Attentional biases may be adaptive for recognizing rewards, but they can become maladaptive and have been implicated in addictive behaviors (Anderson, 2021, Field and Cox, 2008). Understanding these cognitive underpinnings is vital for creating models of addictive behaviors and devising better interventions or training strategies.

Existing research assessing value-modulated attentional capture (VMAC) effects use behavioral tasks that display target stimuli (e.g., diamonds) and distractors (e.g., circles). Typically, the color of the distractors signals the amount of reward (e.g., points) available for participants, with high-value distractors indicating that one may win more points. As shown in various studies (e.g., Albertella et al., 2017), participants tend to look at high-value distractors even if doing so is counterproductive. Typically, increased VMAC scores reflect heightened attentional capture, signifying a more pronounced impact of reward cues on attention. Several studies point to positive associations between VMAC scores and indices of psychopathology. For instance, higher VMAC scores were shown to be associated with higher scores for obsessive–compulsive behaviors (Albertella, Le Pelley, et al., 2019) and prospectively assessed non-abstinence from alcohol (Albertella et al., 2021). Other studies however did not find an association between VMAC effects and alcohol- and cannabis-related problems (Albertella et al., 2017, Freichel et al., 2023). Less is known about the relationship between VMAC and non-substance addictive behaviors, such as addictive-like eating behavior and problematic smartphone use. Our study aimed to primarily test the association between VMAC and substance-use-related problems among an online sample of heavy and light drinkers. In addition, we also investigated the association between VMAC and non-substance addictive behaviors.

Automatic attentional capture effects may be influenced by more reflective processes and individuals’ general cognitive control. To measure cognitive control, researchers have typically employed two distinct methods: 1) self-report questionnaires that instruct individuals to evaluate their perceived level of cognitive control in different situations, and 2) behavioral tasks designed to assess cognitive control based on performance (e.g., speed and accuracy). Thus far, investigators have primarily examined the role of behavioral measures of cognitive control in the interplay between VMAC and psychopathology. For instance, selective attention moderated the association between VMAC and illicit substance use (Albertella et al., 2017). Illicit substance use was associated with greater value modulated attentional capture only in individuals with lower levels of cognitive control. This pattern of cognitive control functions moderating the relationship between implicit, automatic associations and behaviors has been found in many domains (Grenard et al., 2008, Thush et al., 2008, Wiers et al., 2010). Following this potential interaction between cognitive control and attentional capture, we predicted that greater attentional capture of stimuli signaling reward (indexed by the VMAC score) will be associated with more substance-use related problems and addictive behaviors in individuals with low cognitive control. Related to this task-based assessment of attentional bias, our goal was to examine the association between self-reported cognitive control and substance use-related problems and addictive behaviors, and the potential moderating role of self-reported cognitive control. As described by Dang et al. (2020), behavioral and self-report measures tend to be weakly correlated as they assess distinct cognitive response patterns (i.e., structural situational responses to stimuli compared with individuals’ subjective perception of performance).

A barrier to neurocognitive assessment is the arduous nature of traditional task paradigms. Cognitive tasks have dull/unimaginative visual displays and often take a long time to complete which can result in boredom, low effort, and subsequently impact task performance (DeRight & Jorgensen, 2015). Gamification has been proposed as a potential solution to boost motivation during cognitive assessments (Lumsden et al., 2016), and gamified cognitive tasks have been shown to be more engaging and maintain positive affect during the course of a task (Bernecker & Ninaus, 2021). This is particularly important as an engaging gamified VMAC task may enable self-administration, and thus provide an accessible and scalable tool that can be used on online crowdsourcing/data collection platforms. The present study used a novel gamified VMAC task paradigm (i.e., in the context of a football game, Lee et al., 2023) in an online community sample consisting of individuals with varying levels of alcohol use. This recruitment strategy targeting an online community sample was considered appropriate as the task has been validated previously in a Mechanical Turk online sample (Lee et al., 2023).

The present study has three main aims: First, following our pre-registration, we aimed to examine whether this novel gamified VMAC version is suitable for assessing attentional capture effects; and if so, we aimed to examine the association between VMAC and substance use-related problems and non-substance-related addictive behaviors. Second, we aimed to examine whether a behavioral measure of cognitive control (i.e., Stroop Deadline Task) would moderate this relationship. Third, in an exploratory fashion, we aimed to examine the association between a self-reported measure of cognitive control (i.e., WebExec measure of problems with executive functions) and substance use-related and non-substance-related problems. Moreover, we aimed to examine whether this self-reported measure of cognitive control moderates the association between VMAC and substance use-related problems.

## 2 Materials and methods

We preregistered the variable selection, data analysis plan, and predictions for both substance use (https://osf.io/c6prv/?view_only = a08c696f7ec448628e80b8497746f16d) and addictive behaviors (https://osf.io/9epnx/?view_only = f9a24ce8d254457cb19674c557188674) on the Open Science Framework (OSF) before starting data collection.

### 2.1 Procedure

We recruited 300 participants in total, targeting individuals with low to medium alcohol use (0–14 drinks/week, target *n* = 150) and individuals with heavy alcohol use (14 + drinks/week, target *n* = 150). This recruitment strategy was considered appropriate as we were primarily interested in studying associations between VMAC effects and problematic alcohol use. Recruitment took place through the platform *Prolific* (an online platform for recruitment of participants for paid research studies) which allowed us to recruit participants located across Europe. After providing informed consent and basic demographic information on the online platform *Qualtrics*, all participants completed the two cognitive tasks, namely the gamified VMAC task and the Stroop Adaptive Deadline Task (SDL). The task order was randomized across participants. Participants subsequently completed the self-report measures. The entire study session lasted approximately 30 min and participants were reimbursed for their participation. The entire session was done online via laptop or desktop computer and in English. The eligibility criteria included: 18–60 years of age; English proficiency; not color blind and no diagnosis of a neurological condition (e.g., stroke, brain injury, dementia). The study received ethical approval at the Psychology Department of the University of Amsterdam (2022-DP-15645).

## 3 Measures

### 3.1 Value-Modulated attentional capture (VMAC) task

**Fig. 1:**
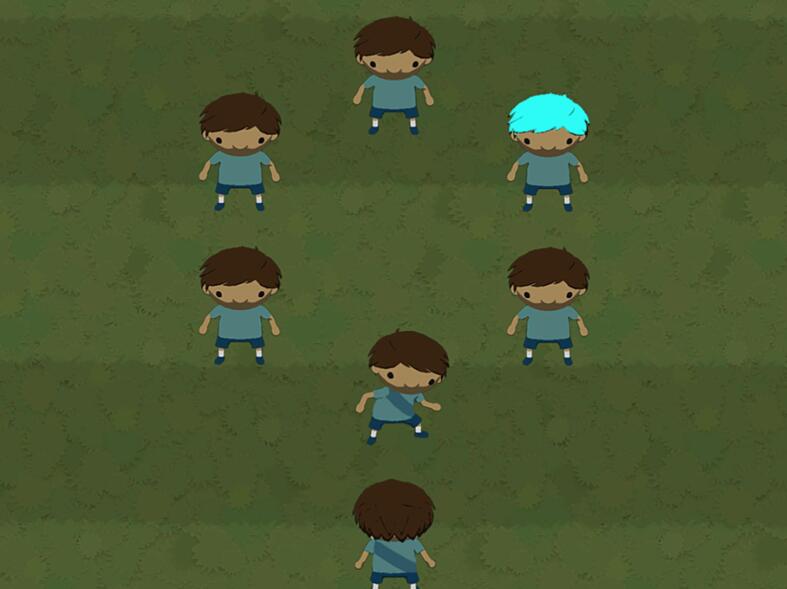
VMAC task trial Note: The color of the opposition player’s hair (blue) signifies the reward value of the trial. The player (bottom center of screen) must pass the ball to their teammate as fast as possible (to the right). (For interpretation of the references to color in this figure legend, the reader is referred to the web version of this article.)

The BrainPAC Value-Modulated Attentional Capture Task (VMAC, Lee et al., 2023) was used to measure reward-related attentional capture. This task is a gamified version of the original VMAC task (Albertella et al., 2019a, Le Pelley et al., 2015) and follows a soccer game format (see Fig. 1). During each trial, a circle of soccer players, including one teammate and 5 opponents (distinguished by jersey patterns), appears. The participant must pass the ball (via left or right button press) as fast as they can to their teammate. The faster they correctly pass the ball, the more points they can earn. Players are instructed that on some trials, one of two distractors are present: one of the opposition players will have one of two hair colors, signaling the magnitude of the reward that may be won on that trial. The high-value hair colour (distractor) signifies the potential to earn ten times the points of whatever would be earned for the same response time with a low-value distractor (hair colour). The points earned per trial are calculated according to the speed at which the player passes the ball. The VMAC has 5 blocks of 24 trials, 10 per distractor type and 4 trials with no distractors present. Test trials were preceded by 6 practice trials; once 50 % accuracy on the practice trials was achieved, the player could then commence the task. The VMAC score is calculated by subtracting the reaction time (RT) on correct low-value distractor trials from correct high-value distractor trials. An accuracy score is calculated by subtracting incorrect passes on low-value from high-value distractor trials. The primary outcome metric of the task is the VMAC score (reaction time) on the last block of the task, with higher scores indicating more reward-related attentional capture. A validation study by Lee et al. (2023) showed that indices from this gamified VMAC version correlate significantly with the standard non-gamified version but show poor test–retest reliability.

### 3.2 Stroop Deadline task

The Stroop Adaptive Deadline Task (Burgoyne & Engle, 2020) was used as a general measure of attentional control. Participants were instructed to respond to the color of the target word displayed on the screen while ignoring the meaning of the word. This version of the task included a response deadline that was adapted to participants’ performance. The response deadline got shorter as participants’ accuracy increased. Participants’ individual response deadline after the last (18th) block was used as the SDL outcome score. Lower SDL scores indicated better attentional control. The adaptive deadline task showed high test–retest reliability and is described in more detail elsewhere (Burgoyne and Engle, 2020, Freichel et al., 2023).

### 3.3 Problematic alcohol and cannabis use

The Alcohol Use Disorder Identification Test (AUDIT; Saunders et al., 1993) is a recognized scale measuring the frequency and harm of alcohol use over 12 months. The Cannabis Use Disorders Identification Test-Revised (CUDIT; Adamson & Sellman, 2003) assesses the frequency and harm of cannabis use over six months through a 10-item survey. Sum scores range from 0 to 40, with higher scores indicating more alcohol or cannabis-related issues. We have calculated Cronbach’s alpha as a measure of internal consistency for all clinical self-report measures (see Table S1 in the supplementary materials).

### 3.4 Addictive behaviors

The modified version of Yale Food Addiction Scale 2.0 (mYFAS; Schulte & Gearhardt, 2017) consists of 13 items that assess eating habits in the past 12 months. The measure includes indicators for the 11 DSM-5 criteria for substance-use disorders as well as associated distress and impairment. A recent psychometric review of the mYFAS 2.0 indicated high internal reliability and factorial validity (Meule & Gearhardt, 2019). The short-version of the Smartphone Addiction Scale (SAS-SV; Kwon et al., 2013) is a 10-item measure of problematic smartphone use with good predictive and convergent validity (Andrade et al., 2020). The SAS-SV assesses the frequency of problematic smartphone use-related symptoms during the past 12 months, such as loss of control and preoccupation.

### 3.5 Self-reported executive functioning problems

The WebExec measure of problems with executive functions (Buchanan et al., 2010) was used to assess self-reported levels of executive functioning (EF). This measure has been validated for online use with non-clinical populations and it showed good construct (Buchanan et al., 2010) and convergent validity (Magis-Weinberg et al., 2020). The short questionnaire consisted of six items on a four-point response scale (1 = *No problems experienced*, 4 = *A great many problems experienced*) assessing different executive functioning problems. An example item is “Do you find yourself having problems concentrating on a task?”. We calculated a total sum score (WebExec score, range: 6–24) with higher numbers indicating more executive functioning problems.

### 3.6 Data analysis

After removing outliers (*n* = 15, based on preregistered VMAC accuracy criteria and one subject with implausible values), our final sample size was 285. To test the presence of attentional bias for high reward and to examine how this bias may change over trials, we analyzed both the reaction time and accuracy measures of the VMAC task using separate 5 x 2 repeated measures analyses of variance (rm-ANOVAs) with block (1–5) and distractor type/condition (high reward, low reward) as within-subject factors.

We analyzed associations between addictive behaviors (AUDIT, CUDIT, mYFAS, SAS-SV) and VMAC scores using separate regression models. We used multiple regression to see how the SDL score, a cognitive control measure, might moderate these associations. These models considered VMAC scores, SDL scores, and their interaction as predictors. Analogous to the moderation analyses using the SDL score, we have used separate regression models to examine how the WebExec score may moderate the associations between addictive behaviors and VMAC scores. In an exploratory fashion, we also examined associations between the WebExec Buchanan measure of executive functioning and addictive behaviors using multiple regression. All models were adjusted for age and sex. Due to the highly skewed and zero-inflated distribution of the mYFAS symptom count measure, we have used zero-inflated negative binomial regression models for this measure.

## 4 Results

### 4.1 Sample descriptives

Important sample characteristics can be found in Table 1. The sample (*n* = 285) was predominantly (72 %) male and showed substantial variability with respect to substance use and behavioral addictions. About half of the participants (53.89 %) showed hazardous or harmful alcohol use (i.e., AUDIT score ≥ 8). Less than one third (29.63 %) of individuals scored above the cutoff (CUDIT score ≥ 9) for cannabis use disorder.

**Table 1:** Sample Characteristics.

**Category**	**Level**	**Proportions (n)**
Education	Advanced degree Bachelor/Associate degree	17 % (47) 42 % (1 1 6)
	High school degree	40 % (1 1 0)
	Below high school degree	1 % (3)
Gender	Man	72 % (1 9 8)
	Woman	25 % (69)
	Non-binary / gender diverse/ other	3 % (8)
Sex	Male	72 % (1 9 9)
	Female	27 % (74)
	Prefer not to say	1 % (3)
**Category**	**Mean**	**SD**
Age	33.17	12.26
AUDIT Total Score	8.95	6.32
CUDIT Total Score	6.48	5.78
WebExec Score	11.83	4.23
SDL Response Window (ms)	924.63	264.15
mYFAS Symptom Count	0.76	1.71
SAS Total Score	24.78	10.04

*Note.* n = number of individuals. The *n* slightly differs across the variables due to missing responses on certain measures. AUDIT = Alcohol Use Disorder Identification Test, CUDIT = Cannabis Use Disorders Identification Test, VMAC = Value-Modulated Attentional Capture, SDL = Stroop Adaptive Deadline Task, mYFAS = modified version of Yale Food Addiction Scale 2.0, SAS = Smartphone Addiction Scale, SD = Standard Deviation, ms = milliseconds. WebExec score refers to the measure of self-reported problems with cognitive functions. The gender category “other” refers to gender identities not listed in the present survey. Advanced degrees refer to Master’s degrees, Doctorates, and Ph.D.

### 4.2 Gamified VMAC effects

**Fig. 2:**
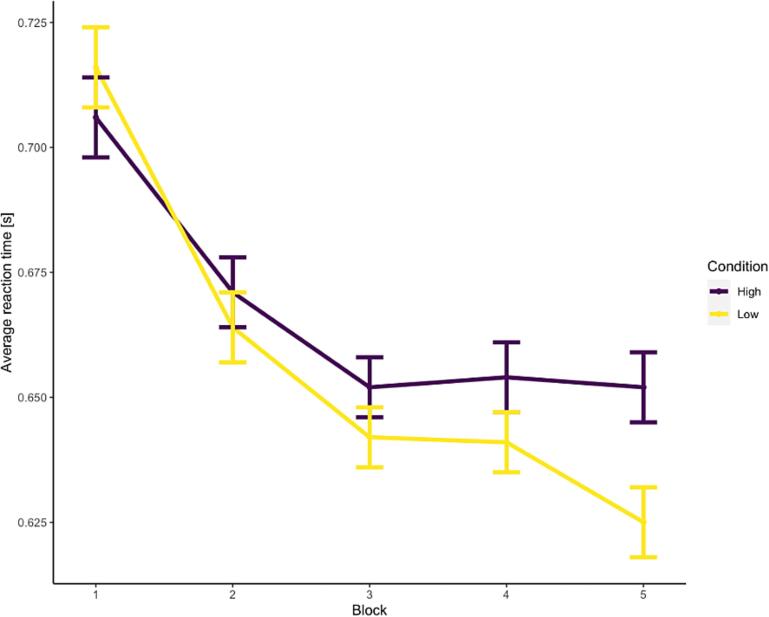
VMAC Effect Across Blocks *Note.* High and low refers to the value of distractors (*high* = high-value distractors, *low* = low-value distractors). Standard errors are shown in vertical bars. *s* = seconds.

***Reaction times.***Fig. 2 shows reaction times for different task conditions (high vs. low reward) and blocks (1–5). RTs between different blocks were strongly correlated with each other (i.e., average correlation: 0.61 for low-reward, and 0.57 for high-reward). A significant difference between high and low rewards appeared after the third block of the task, with significantly higher reaction times for high reward compared with low reward conditions (see Table S2). Consistent with Fig. 2, we found significant main effects of both condition (*p* <.01) and block (*p* <.01). Participants responded significantly faster in trials with low-reward distractors, and they responded faster as they completed more blocks. We found a significant interaction between condition and block, reflecting the increasing difference between high and low reward trials across the 5 blocks.

***Accuracy.*** To test for potential speed-accuracy tradeoff effects, we examined the accuracy per block and condition (see Figure S1). Our analysis revealed no significant main effect of condition (*p* =.87) on test accuracy. We found a significant main effect of block (*p* <.01), indicating that participants responded more accurately the more blocks they completed. This effect was qualified by a significant interaction between block and condition (*p* < 0.01) indicating that the effect of block differed between both conditions. It appeared that particularly during block 4, participants in the low reward condition were more accurate compared to the high reward condition (see Table S2).

### 4.3 Associations between gamified VMAC, general cognitive control, and addictive behaviors

None of the regression models were significant. VMAC score was not significantly associated with either AUDIT (*p* =.16), CUDIT (*p* =.11), mYFAS (*p* =.22), or smartphone-related problems (*p* =.38). See Tables S4-S7 for an overview of all test statistics. The SDL score was also not significantly associated with VMAC (*p* =.23), AUDIT (*p* =.48), CUDIT scores (*p* =.77), mYFAS (*p* =.44), and smartphone-related problems (*p* =.31). For the interaction analysis (see Tables S8-11), our regression models showed no significant interaction effects (between VMAC and SDL score) for the AUDIT total score (*p* =.71), CUDIT total score (*p* =.40), mYFAS (*p* = 0.12), and smartphone-related problems (*p* = 0.47).

### 4.4 Exploratory analysis of self-reported EF problems and symptom measures

As an exploratory extension of our main analysis, we investigated the association between self-reported EF problems and the symptom measures.

**Fig. 3:**
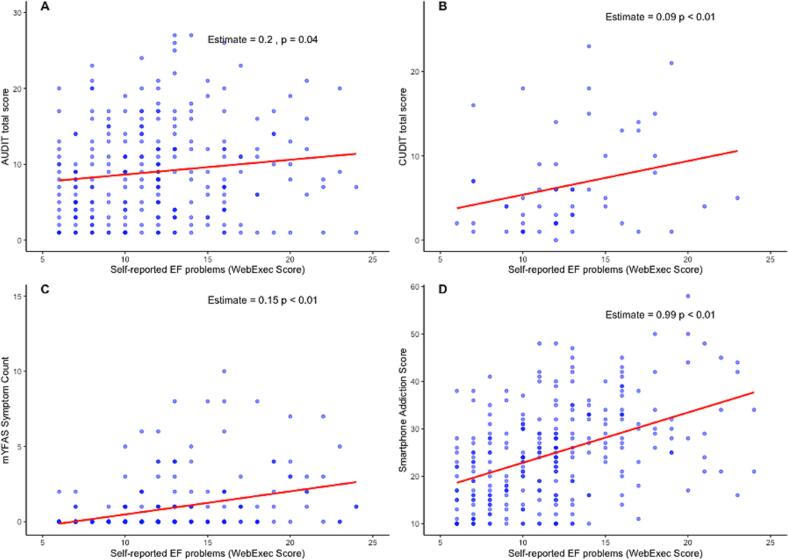
Association Between the WebExec Measure of Executive Functioning Problems and Symptom Measures *Note.* The estimates refer to the main effects (beta-estimates) of the self-reported EF problems measure in the respective regression model. All models also included sex and age as covariates. A higher color saturation indicates a higher frequency of scores in the figure. A zero-inflated negative binomial regression model to test the association between mYFAS symptom count and the self-reported EF problems measure showed a similar (significant) relationship. EF = executive functions, AUDIT = Alcohol Use Disorder Identification Test, CUDIT = Cannabis Use Disorders Identification Test, mYFAS = modified version of Yale Food Addiction Scale 2.0.

We found that this self-reported measure of problems with executive functions was significantly associated (see Fig. 3) with more alcohol-related problems (AUDIT total score), cannabis-related problems (CUDIT total score), addictive-like eating behavior (mYFAS symptom count), and smartphone-related problems (see Table S12 for all statistics). More problems with executive functions were associated with more substance-use related problems and addictive behaviors. The WebExec score was not significantly (*p* > 0.05) associated with either the VMAC or the SDL score.

**Fig. 4:**
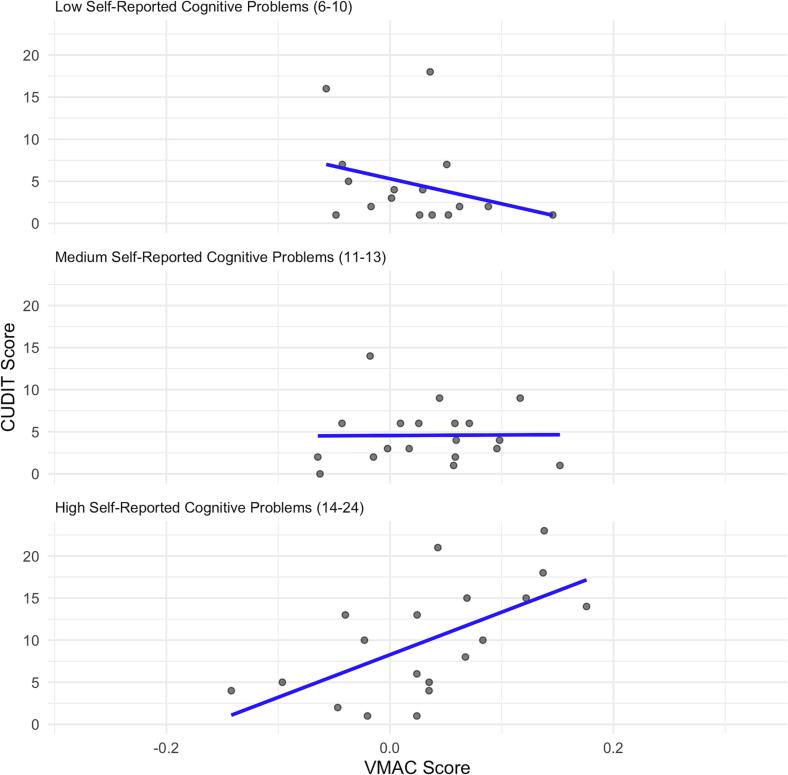
Association between VMAC Score and CUDIT At Different Levels of Self-reported Cognitive Problems *Note.* VMAC = Value-Modulated Attentional Capture; CUDIT = Cannabis Use Disorders Identification Test. WebExec score refers to the measure of self-reported problems with cognitive functions. The grouping (low, medium, high) was based on the WebExec scores falling within these percentile cutoffs (33rd 66th percentiles).

In addition, we conducted exploratory analyses that examined whether this self-reported measure of problems with executive functions would moderate the associations between VMAC scores and all substance use-related problems and addictive behaviors. We found a significant positive interaction effect for cannabis use-related problems (*p* = 0.04). Among individuals with higher levels of self-reported cognitive problems (see Fig. 4), higher VMAC scores (i.e., stronger attentional capture) were associated with more cannabis use-related problems. None of the interaction effects for the other outcome measures were significant (*p* > 0.05, see Table S13 for all test statistics).

## 5 Discussion

The study aimed to: 1) evaluate a gamified VMAC task for assessing reward-driven attentional capture, 2) explore its correlation with both substance and non-substance addictive behaviors, and 3) determine whether both behavioral and self-reported measures of cognitive control moderate these relationships. We found consistent VMAC effects that were, however, not significantly associated with either substance use, or non-substance use-related addictive behaviors. A behavioral measure of general cognitive control did not moderate the association between VMAC and alcohol-/cannabis-related problems or behavioral addictions. Exploratory analyses revealed that 1) a short self-report measure of problems with cognitive functions was associated with both substance use-related problems and addictive behaviors, and 2) this measure of cognitive control moderated the association between VMAC and cannabis use-related problems.

### 5.1 Potential of gamification in attentional bias research

Our results indicate that a novel gamified version of the traditional VMAC task was effective in capturing attentional biases towards high-reward stimuli that were established in prior work (Pearson et al., 2022). Participants attended more to the distractor that signaled a high-value reward compared with a low-value reward - an effect that was present already after the third block during the task. The present study is one of the first (also see Lee et al., 2023) to show such effects in a gamified version of the VMAC task. Future studies should include both the non-gamified as well as the gamified VMAC task to evaluate differences with respect to participant engagement, task performance, and the reliability of the cognitive assessment. Considering the increasing popularity of scalable online cognitive task assessment in research, an engaging gamified VMAC task with good psychometric properties may present a future tool for applied researchers.

### 5.2 Reward-related attentional capture and addictive behaviors

Contrary to our predictions, we did not find a significant association between reward-related attentional capture and any of the four addictive behaviors. Despite strong theoretical rationale that problematic alcohol use would be associated with heightened reward-related attentional bias, this has not been evidenced empirically (Albertella et al., 2017, Albertella et al., 2019b, Freichel et al., 2023). In fact, our findings replicate those of Albertella et al., 2019a, Albertella et al., 2021, Albertella et al., 2019b) who failed to find a relationship between problematic alcohol use and VMAC performance. This is possibly related to the strong influence of motivation on self-regulation of drinking behavior (Kopetz et al., 2013). For instance, the VMAC score has been shown to be associated with more compulsive drinking but in the opposite direction when an individual’s primary motivation to drink is relief-based (Liu et al., 2021). Meaning, that individuals with more goal-directed performance on the VMAC (i.e., consistently following task instructions) also engage in alcohol use in a goal-directed manner (i.e., to relieve distress). Our study did not capture such motivational profiles and thus, it is possible that only compulsive alcohol use may be associated with attentional capture. We also failed to find a relationship between VMAC performance and addictive-like eating behavior or problematic smartphone use. This is contrary to previous work that has shown more severe “food addiction” is associated with a heightened attentional bias towards reward cues (Adams et al., 2019). However, it may be due to the nature of our sample, which mostly showed no/low symptoms of addictive-like eating behavior. In terms of problematic smartphone use, despite our sample having varied severity of use, we also did not find a relationship between VMAC performance and problematic smartphone use. This was contrary to what was expected given previous literature linking attentional bias to problematic use of the internet/social networking sites (Nikolaidou et al., 2019). However, most prior work focused on attentional bias toward social media-specific cues rather than general reward cues as presented in the present VMAC task.

Moreover, prior studies have shown that variations in levels of cognitive control among individuals may account for the likelihood of automatic attentional processes being related to addictive behaviors (Houben & Wiers, 2009). Our findings did not support this hypothesis that greater attentional capture (indicated by the VMAC score) will be associated with higher AUDIT and CUDIT total scores in respondents with weak general cognitive control (SDL response window). An exploratory extension of the study found no such moderation effects for addictive behaviors (addictive-like eating behavior and problematic smartphone use). The null finding for alcohol-related problem based on the behavioral measure is consistent with previous reports showing no interaction effects for alcohol-related problems but instead only for anxiety symptoms (Freichel et al., 2023). However, exploratory analyses revealed that attentional capture was indeed associated with more cannabis use-related problems only among individuals with a high level of self-reported problems with cognitive functions. This is analogous to the study by Albertella et al. (2017) that showed an association between illicit substance use and greater value-modulated attentional capture in individuals with lower levels of cognitive control. Our finding may suggest that attentional mechanisms might only drive substance use-related problems in people with high subjective cognitive problems. However, considering the cross-sectional nature of our study and low number of individuals with heavy cannabis use, there may be alternative explanations for this finding: 1) A combination of cognitive problems and attentional capture may drive cannabis use-related problems, 2) a combination of cannabis use-related problems and cognitive problems may cause attentional capture, 3) a combination of cannabis use-related problems and attentional capture may cause cognitive problems, or 4) other unmeasured factors may explain the co-occurrence of higher cannabis use-related problems, attentional capture, and cognitive problems. Further longitudinal research among broader samples (including non-users before initiation and heavy cannabis users) is necessary to determine whether attentional capture and cognitive problems precede, co-occur, or lag behind substance use-related problems.

The apparent inconsistency in findings between behavioral and self-report measures may be due to our use of a novel SDL task to assess cognitive control. It is possible that the index extracted from the SDL task does not reflect differences in general cognitive control. In this task, the adaptive response window after the last block is used as an indicator of general cognitive control. While this task showed high test–retest reliability, it may also conflate differences in motivation and performance throughout the blocks that impact individuals’ final adaptive response window. Thus, future studies should further investigate whether other tasks assessing general cognitive control may indeed moderate the relationship between attentional biases and psychopathology.

### 5.3 Associations between self-reported problems with cognitive functions and substance use-related problems and addictive behaviors

An exploratory extension of our preregistered study was to investigate associations between self-reported problems with cognitive functions and substance use related problems and addictive behaviors. Interestingly, we found that more self-reported problems with cognitive functions were associated with more alcohol-, and cannabis-related problems as well as with higher scores on a variety of addictive behaviors, including symptoms of possible non-substance addictions (food and problematic smartphone use). These results highlight the transdiagnostic value of perceived problems with cognitive functioning for substance use-related problems and addictive behaviors. This is in line with evidence in favor of the ‘C’ (cognitive dysfunction)-factor (Abramovitch et al., 2021) as cognitive dysfunction may represent a transdiagnostic dimension underlying psychopathology (Goschke, 2014). It is important to note that the self-report measure of problems with executive functions (WebExec score) was not significantly associated with task-based estimates of attentional capture (VMAC) or general cognitive control (SDL window). This is consistent with prior work indicating low correlations between task performance and self-report as these sources may tap into distinct abilities or windows of cognitive control (Dang et al., 2020, Snyder et al., 2021, White et al., 1994). Likely, both self-reported levels of symptoms as well as self-reported problems with cognitive functions share variance due to similar response biases in participants’ self-report. Although more work is needed to understand the mechanism underlying these associations, our study is the first to show such broad cross-construct associations of self-reported cognitive problems with different substances and addictive behaviors.

## 6 Limitations and conclusions

There are several important limitations that should be noted. First, we used a novel gamified version of the traditional VMAC task. Given its novelty, existing research on the task and its psychometric properties is still in its infancy. While our findings showed evidence for its effectiveness in capturing value-modulated attentional bias, more validation studies are necessary to investigate its reliability and validity. Second, participants completed the study entirely online for financial reimbursement. While such online data collection effort allows researchers to target a more diverse participant pool and specific groups (i.e., heavy drinkers) rather than usual college student samples, it is possible that participants were not fully engaged during the completion of the cognitive task. We have used attention check items and post-hoc data quality checks to mitigate these concerns.

In conclusion, our study is one of the first to provide preliminary evidence for the utility of gamified versions of the VMAC task in capturing attentional biases towards reward stimuli. However, such indices of attentional bias were not associated with substance-use related problems or addictive behaviors. Our findings highlight the transdiagnostic value of assessing self-reported problems with cognitive functions for substance use-related problems and addictive behaviors, yet more work is needed to understand the underlying mechanisms. The assessment of attentional capture effects using an engaging online task is still in its nascent stages. If successful, this would pave the way for easy, scalable, and engaging assessment of attentional capture.

## Role of Funding Source

This study is part of the project ‘New Science of Mental Disorders’ (https://www.nsmd.eu), supported by the Dutch Research Council and the Dutch Ministry of Education, Culture and Science (NWO gravitation grant number 024.004.016).

## CRediT authorship contribution statement

**René Freichel:** Writing – review & editing, Writing – original draft, Visualization, Methodology, Investigation, Formal analysis, Conceptualization. **Erynn Christensen:** Writing – review & editing, Visualization, Methodology, Investigation, Formal analysis, Conceptualization. **Lana Mrkonja:** Writing – review & editing, Methodology, Investigation, Conceptualization. **Peter J. de Jong:** Writing – review & editing, Supervision, Conceptualization. **Janna Cousijn:** Writing – review & editing, Supervision, Conceptualization. **Ingmar Franken:** Writing – review & editing, Supervision, Conceptualization. **Murat Yücel:** Writing – review & editing, Supervision, Conceptualization. **Rico Lee:** Writing – review & editing, Supervision, Conceptualization. **Ilya M. Veer:** Writing – review & editing, Supervision, Conceptualization. **Lucy Albertella:** Writing – review & editing, Supervision, Methodology, Investigation, Conceptualization. **Reinout W. Wiers:** Writing – review & editing, Supervision, Methodology, Investigation, Funding acquisition, Conceptualization.

## Declaration of competing interest

The authors declare the following financial interests/personal relationships which may be considered as potential competing interests: Murat Yücel receives funding from: government funding bodies such as the NHMRC, Australian Research Council (ARC), Australian Defence Science and Technology (DST), the Department of Industry, Innovation and Science (DIIS), the National Institutes of Health (NIH, USA); philanthropic donations from the David Winston Turner Endowment Fund, Wilson Foundation; sponsored Investigator-Initiated trials including Incannex Healthcare Ltd; and payments in relation to court-, expert witness-, and/or expert review-reports. Murat Yücel also sits on the Advisory Boards of: Centre of The Urban Mental Health, University of Amsterdam; Monash Biomedical Imaging Centre; and Enosis Therapeutics. These funding sources had no role in the data analysis, presentation, or interpretation and write-up of the data. All other authors declare no conflicts of interest.

## Data Availability

Data will be made available on request.
