# Supplementary material for: Attentional Biases and Their Association with Substance-Use-Related Problems and Addictive Behaviors: The Utility of a Gamified Value-Modulated Attentional Capture Task
Source: Addict Behav Rep. 2024 Feb 13;19:100534. doi: 10.1016/j.abrep.2024.100534 (PMC10885317; doi:10.1016/j.abrep.2024.100534)
Supplement: Supplementary data 1 [file mmc1.docx]

**Attentional Biases and Their Association with Substance-Use-Related Problems and Addictive Behaviors: The Utility of a Gamified Value-Modulated Attentional Capture Task**

**Supplementary Materials**

[**Supplementary Materials Section 1: Statistical Analysis and Cronbach’s Alpha** 2](#_Toc157078036)

[**Supplementary Materials Section 2: VMAC Effects** 4](#_Toc157078037)

[**Supplementary Materials Section 3: VMAC - Clinical Associations** 7](#_Toc157078038)

[**Supplementary Materials Section 4: VMAC - Stroop Interaction Effects** 9](#_Toc157078039)

[**Supplementary Materials Section 5: Self-reported Executive Functioning Problems - Clinical Associations** 11](#_Toc157078040)

# **Supplementary Materials Section 1: Statistical Analysis and Cronbach’s Alpha**

**Outlier exclusion**

We excluded 14 individuals based on the preregistered value-modulated attentional capture (VMAC) accuracy criteria (i.e., less than 55% accuracy in the test phase of the VMAC task) and one participant because of implausible (i.e., extreme values) data. The attention check used was a single item in which participants were instructed to select the term “Lavender note” out of a range of phonetically similar terms (lavender quote/vote/note/tote). All 285 participants successfully completed the attention check (i.e., selecting the term “Lavender note” out of lavender quote/vote/note/tote) in Qualtrics, and did not meet any of the other preregistered outlier criteria for the VMAC (i.e., too fast responses) or the Stroop Adaptive Deadline Task (SDL) (e.g., accuracy lower than 70% during practice phase) tasks.

**Statistical analysis**

For each addictive behavior, only individuals who had ever engaged in the behavior before (i.e., consumption of alcohol, cannabis, possession of smartphone) were included in the respective regression model.

**Cronbach’s alpha**

**Table S1**

*Cronbach’s Alpha for all clinical measures*

|  | Cronbach‘s Alpha |
| --- | --- |
| AUDIT total score | .82 |
| CUDIT total score | .80 |
| WebExec score | .89 |
| mYFAS symptom count | .86 |
| SAS score | .89 |

*Note.* AUDIT = Alcohol Use Disorder Identification Test, CUDIT = Cannabis Use Disorders Identification Test, WebExec score= self-reported problems with cognitive functions; mYFAS = modified version of Yale Food Addiction Scale 2.0; SAS = Smartphone Addiction Scale.

# **Supplementary Materials Section 2: VMAC Effects**

**VMAC reaction-time analysis**

**Table S2**

*ANOVA results for VMAC reaction-times*

| **Effect** | **DFn** | **DFd** | **F** | **P** |
| --- | --- | --- | --- | --- |
| Condition | 1 | 286 | 18.02 | < .01 |
| Block | 3.13 | 894.29 | 59.107 | < .01 |
| Condition * block | 3.78 | 1080.65 | 6.47 | < .01 |

*Note.* DF = Degrees of Freedom for the numerator (n) and denominator (d). F = F-statistic.

**VMAC accuracy analysis**

The accuracy metric shown in Figure S1 represents the percentage accuracy of the response per set across the task for low compared with high-value distractor trials.

**Figure S1**

*VMAC Accuracy for all blocks and conditions*

**
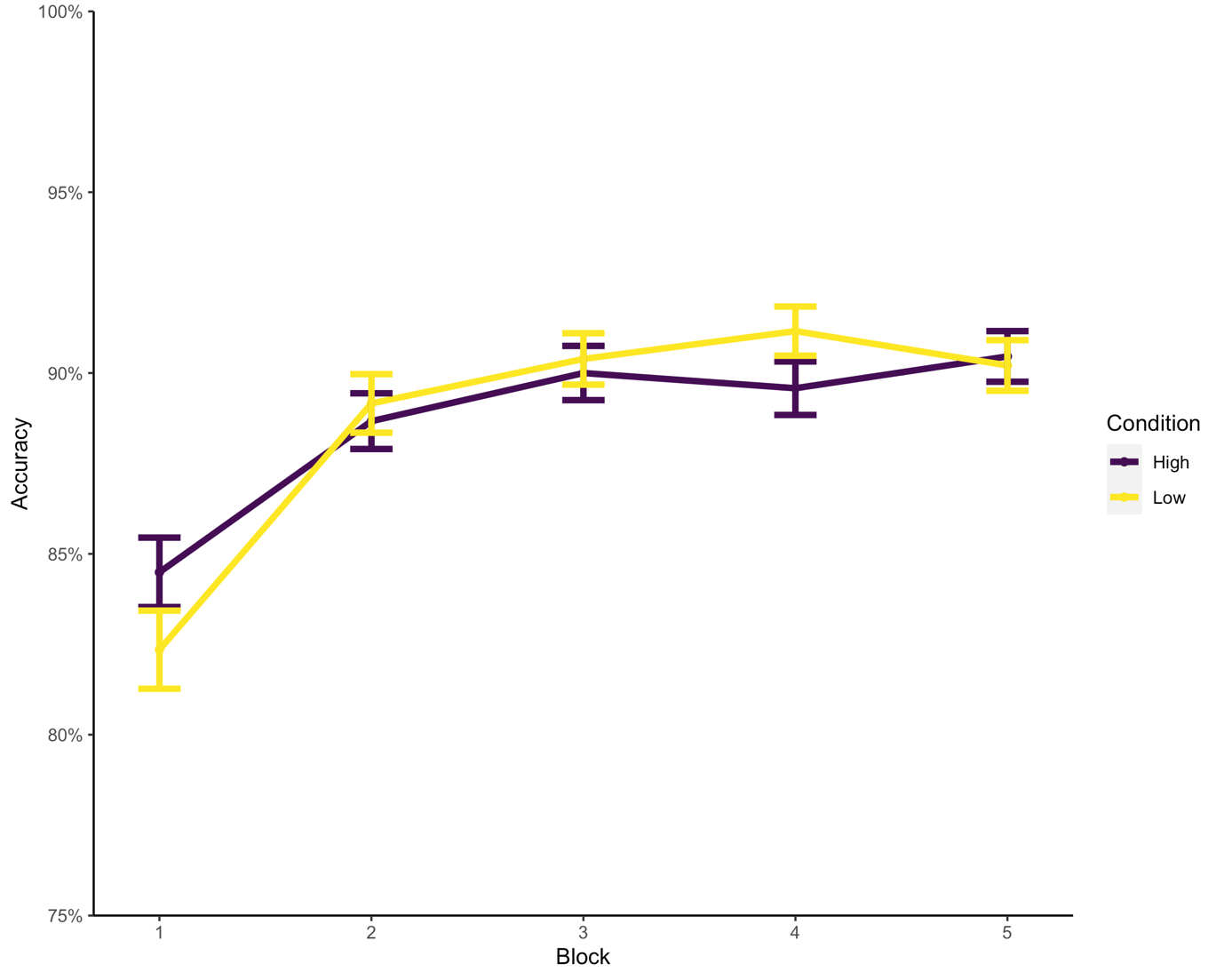
**

**Table S3**

*ANOVA results for VMAC accuracy*

| **Effect** | **DFn** | **DFd** | **F** | **p** |
| --- | --- | --- | --- | --- |
| Condition | 1 | 286 | 0.026 | .872 |
| Block | 2.93 | 836.88 | 40.429 | < .01 |
| Condition * block | 3.88 | 1108.72 | 2.739 | .029 |

*Note.* DF = Degrees of Freedom for the numerator (n) and denominator (d). F = F-statistic.

# **Supplementary Materials Section 3: VMAC - Clinical Associations**

The tables below summarize the results for the regression models in which the VMAC score, sex, and age were included as predictors.

**Table S4**

*Regression results for AUDIT total score*

| **Effect** | **Estimate** | **SE** | **Statistic** | **p** |
| --- | --- | --- | --- | --- |
| Intercept | 5.99 | 0.86 | 6.94 | <.01 |
| VMAC Score | -6.71 | 4.77 | -1.41 | .16 |
| Sex | -0.02 | 0.83 | -0.03 | .98 |
| Age | 0.09 | 0.02 | 3.93 | <.01 |

*Note.* AUDIT = Alcohol Use Disorder Identification Test; VMAC = Value-Modulated Attentional Capture; SE = Standard Error. This analysis included everyone with valid AUDIT scores (n = 258). The dichotomized sex variable includes 'Male' (coded as 0) and 'Female' (coded as 1). The coefficient for Sex indicates the estimated difference between female and male.

**Table S5**

*Regression results for CUDIT total score*

| **Effect** | **Estimate** | **SE** | **Statistic** | **p** |
| --- | --- | --- | --- | --- |
| Intercept | 1.45 | 0.36 | 4.09 | <.01 |
| VMAC Score | 2.98 | 1.86 | 1.61 | .11 |
| Sex | -0.07 | 0.33 | -0.23 | .82 |
| Age | 0.01 | 0.01 | 1 | .32 |

*Note.* CUDIT = Cannabis Use Disorders Identification Test; VMAC = Value-Modulated Attentional Capture; SE = Standard Error. This analysis included everyone with valid CUDIT scores (n = 54). The dichotomized sex variable includes 'Male' (coded as 0) and 'Female' (coded as 1). The coefficient for Sex indicates the estimated difference between female and male.

**Table S6**

*Regression results for mYFAS total score*

| **Effect** | **Estimate** | **SE** | **Statistic** | **p** |
| --- | --- | --- | --- | --- |
| Intercept | 0.97 | 0.43 | 2.23 | .03 |
| VMAC Score | 2.47 | 2.02 | 1.22 | .22 |
| Sex | 0.38 | 0.31 | 1.23 | .22 |
| Age | -0.02 | 0.01 | -1.61 | .11 |

*Note.* mYFAS = modified version of Yale Food Addiction Scale 2.0; VMAC = Value-Modulated Attentional Capture; SE = Standard Error. The dichotomized sex variable includes 'Male' (coded as 0) and 'Female' (coded as 1). The coefficient for Sex indicates the estimated difference between female and male.

**Table S7**

*Regression results for smartphone use total score*

| **Effect** | **Estimate** | **SE** | **Statistic** | **p** |
| --- | --- | --- | --- | --- |
| Intercept | 23.85 | 0.76 | 31.34 | <0.01 |
| VMAC Score | -7.07 | 7.96 | -0.89 | .38 |
| Sex | 4.55 | 1.36 | 3.35 | <0.01 |
| Age | <0.01 | <0.01 | -0.97 | .33 |

*Note.* VMAC = Value-Modulated Attentional Capture. The smartphone use total score was based on the Smartphone Addiction Scale; SE = Standard Error. The dichotomized sex variable includes 'Male' (coded as 0) and 'Female' (coded as 1). The coefficient for Sex indicates the estimated difference between female and male.

# **Supplementary Materials Section 4: VMAC - Stroop Interaction Effects**

**Table S8**

*Regression results for AUDIT total score*

|  | **Estimate** | **SE** | **Statistic** | **p** |
| --- | --- | --- | --- | --- |
| Intercept | 10.12 | 1.53 | 6.62 | <.01 |
| VMAC Score | -9.4 | 17.69 | -0.53 | .60 |
| SDL Score | <0.01 | <0.01 | -0.77 | .44 |
| Sex | 0.21 | 0.90 | 0.24 | .81 |
| Age | <0.01 | <0.01 | -0.41 | .68 |
| VMAC Score * SDL Score | 0.01 | 0.02 | 0.38 | .71 |

*Note.* AUDIT = Alcohol Use Disorder Identification Test; VMAC = Value-Modulated Attentional Capture; SDL = Stroop Adaptive Deadline Task; SE = Standard Error. The dichotomized sex variable includes 'Male' (coded as 0) and 'Female' (coded as 1). The coefficient for Sex indicates the estimated difference between female and male.

**Table S9**

*Regression results for CUDIT total score*

|  | **Estimate** | **SE** | **Statistic** | **p** |
| --- | --- | --- | --- | --- |
| Intercept | 2.44 | 4.26 | 0.57 | .57 |
| VMAC Score | -12.92 | 47.8 | -0.27 | .79 |
| SDL Score | <0.01 | <0.01 | 0.73 | .47 |
| Sex | -0.15 | 2.24 | -0.07 | .95 |
| Age | 0.04 | 0.08 | 0.53 | .60 |
| VMAC Score * SDL Score | 0.04 | 0.04 | 0.85 | .40 |

*Note.* CUDIT = Cannabis Use Disorders Identification Test; VMAC = Value-Modulated Attentional Capture; SDL = Stroop Adaptive Deadline Task; SE = Standard Error. The dichotomized sex variable includes 'Male' (coded as 0) and 'Female' (coded as 1). The coefficient for Sex indicates the estimated difference between female and male.

**Table S10**

*Regression results for mYFAS total score*

| **Effect** | **Estimate** | **SE** | **Statistic** | **p** |
| --- | --- | --- | --- | --- |
| Intercept | 0.69 | 0.93 | 0.75 | .46 |
| VMAC Score | -8.09 | 7.45 | -1.09 | .28 |
| SDL Score | <0.01 | <0.01 | 0.38 | .70 |
| Sex | 0.63 | 0.32 | 1.98 | .05 |
| Age | -0.02 | 0.01 | -1.78 | .07 |
| VMAC Score * SDL Score | 0.01 | 0.01 | 1.56 | .12 |

*Note.* mYFAS = modified version of Yale Food Addiction Scale 2.0; VMAC = Value-Modulated Attentional Capture; SDL = Stroop Adaptive Deadline Task; SE = Standard Error. The dichotomized sex variable includes 'Male' (coded as 0) and 'Female' (coded as 1). The coefficient for Sex indicates the estimated difference between female and male. A zero-inflated count data regression model was used.

**Table S11**

*Regression results for SAS total score*

| **Effect** | **Estimate** | **SE** | **Statistic** | **p** |
| --- | --- | --- | --- | --- |
| Intercept | 28.29 | 2.83 | 10.01 | <.01 |
| VMAC Score | 11.27 | 28.34 | 0.4 | .69 |
| SDL Score | <0.01 | <0.01 | 1.01 | .31 |
| Sex | 4.73 | 1.33 | 3.57 | <.01 |
| Age | -0.21 | 0.05 | -4.32 | <.01 |
| VMAC Score * SDL Score | -0.02 | 0.03 | -0.73 | .47 |

*Note.* SAS = Smartphone Addiction Scale; VMAC = Value-Modulated Attentional Capture; SDL = Stroop Adaptive Deadline Task; SE = Standard Error. The dichotomized sex variable includes 'Male' (coded as 0) and 'Female' (coded as 1). The coefficient for Sex indicates the estimated difference between female and male.

# **Supplementary Materials Section 5: Self-reported Executive Functioning Problems - Clinical Associations**

**Table S12**

*Regression results for the association between WebExec scores and VMAC/clinical outcomes/SDL*

| **Outcome** | **Estimate** | **SE** | **t/z value** | **p** |
| --- | --- | --- | --- | --- |
| VMAC | < 0.01 | < 0.01 | 0.23 | .82 |
| AUDIT | 0.20 | 0.10 | 2.07 | .04 |
| CUDIT | 0.09 | 0.03 | 2.86 | <.01 |
| mYFAS | -0.35 | 0.12 | -2.84 | <.01 |
| SAS | 0.99 | 0.13 | 7.39 | <.01 |
| SDL | 0.02 | 0.02 | 1.48 | .14 |

*Note.* VMAC = Value-Modulated Attentional Capture; *AUDIT = Alcohol Use Disorder Identification Test;* CUDIT = Cannabis Use Disorders Identification Test; mYFAS = modified version of Yale Food Addiction Scale 2.0; SAS = Smartphone Addiction Scale; SDL = Stroop Adaptive Deadline Task. We used a negative binomial generalized linear model to examine the association between WebExec score and the CUDIT total score. A zero-inflated count data regression model was used to examine the association between WebExec score and mYFAS symptom count. In both models (for CUDIT and mYFAS), the relevant estimates refer to z-values. All other linear regression model estimates refer to t-values. The estimates for mYFAS refer to the log odds of a count being zero. A negative coefficient suggests that the log odds of the symptom count being zero decrease as self-reported cognitive problems increase.

**Exploratory Moderation Analyses using Self-Reported Executive Functioning Problems**

Following the structure of the models that include the behavioral measure, we conducted four separate regression models (for alcohol-, cannabis-related problems, addictive-like eating behavior, and problematic smartphone use) that include sex, age, the main effects (both VMAC score and WebExec score), and their respective interaction.

Table S13 includes the relevant test statistics for the interaction term (moderation) for all four outcomes. There was a significant positive moderation effect (i.e., interaction WebExec score – VMAC score) on cannabis use-related problems (CUDIT total score). None of the other moderation models were significant (see Table S13).

**Table S13**

*Regression results for moderation analyses including WebExec score and clinical outcomes*

| **Outcome** | **Estimate** | **SE** | **t/z value** | **p** |
| --- | --- | --- | --- | --- |
| AUDIT | 0.81 | 1.03 | 0.79 | .43 |
| CUDIT | 6.16 | 2.94 | 2.09 | .04 |
| mYFAS | -0.71 | 0.44 | -1.61 | .11 |
| SAS | -1.16 | 1.54 | -0.75 | .45 |

*Note. AUDIT = Alcohol Use Disorder Identification Test;* CUDIT = Cannabis Use Disorders Identification Test; mYFAS = modified version of Yale Food Addiction Scale 2.0; SAS = Smartphone Addiction Scale. A zero-inflated count data regression model was used to examine the association between WebExec score and mYFAS symptom count.
